# Decellularized Tumor Tissues Integrated with Polydopamine for Wound Healing

**DOI:** 10.34133/research.0445

**Published:** 2024-08-15

**Authors:** Hongzheng Li, Xiang Lin, Shangrui Rao, Gongting Zhou, Letian Meng, Yunru Yu, Jinglin Wang, Xiaolei Chen, Weijian Sun

**Affiliations:** ^1^ Department of Gastrointestinal Surgery, The Second Affiliated Hospital of Wenzhou Medical University, Wenzhou, 325027, China.; ^2^ Division of Hepatobiliary and Transplantation Surgery, Department of General Surgery, Nanjing Drum Tower Hospital, the Affiliated Hospital of Medical School, Nanjing University, Nanjing, 210008, China.; ^3^ Pharmaceutical Sciences Laboratory, Åbo Akademi University, Turku, 20520, Finland.; ^4^ Department of Gastrointestinal Surgery, The First Affiliated Hospital of Wenzhou Medical University, Wenzhou, 325027, China.

## Abstract

Natural biomaterials have been showing extensive potential in wound healing; attempts therefore focus on productions achieving both antimicrobial and tissue regenerative abilities. Here, we construct a decellularized human colon tumor (DHCT)-derived scaffold for wound remolding via microfluidic bioprinting. The DHCT retains a series of growth factors, fibrin, and the collagen configuration, that favor tissue repair and reconstruction. Specifically, the scaffold shows superior abilities in cell migration and angiogenesis. The biocompatible scaffold is also imparted with tissue adhesion ability and photothermal effect due to the coating of biologically derived polydopamine on the surface. The strong photothermal effect under near-infrared irradiation also present the scaffold with an antibacterial rate exceeding 90%. Furthermore, in vivo experiments convinced that the polydopamine-integrated DHCT scaffold can markedly expedite the healing process of acute extensive wounds. These findings indicate that composite materials derived from natural tumors have substantial potential in pertinent clinical applications.

## Introduction

The high incidence of wounds, especially difficult-to-heal ones, has been causing severe threats to the public healthcare, making it one of the most important social health issues throughout the world [1–3]. Because reduplicative bacterial infection has been recognized as one of the main causes to impede the wound healing, efforts have been devoted to antimicrobial infection while promoting the tissue remolding and regeneration [4–7]. For this purpose, many nanomaterials including silver nanoparticles, oxide nanozyme, and natural nanoparticles have been developed [8–11]. They achieve antibacterial purposes by breaking the bacterial living environment, destroying bacterial membranes, inhibiting DNA replication, and inhibiting enzyme respiration [12–15]. Meanwhile, a myriad of biomedical materials such as polylactic acid, collagen, and hyaluronic acid, have been designed to induce cell proliferation, induce tissue repair, and accelerate wound healing [16–20]. Despite extensive progresses, the generation of these functional materials often experience complicated synthesis or polymerization processes to realize desired biological functions [21–27]. Moreover, although the integration of bioactive or functional reagents will add competing features in wound healing, these exogenous additives could result in suboptimal therapeutic outcomes and thus hinder their applications [28–35]. Therefore, natural bioactive scaffolds with unique antimicrobial properties and tissue remolding capabilities are urgently needed.

**Fig. 1.**
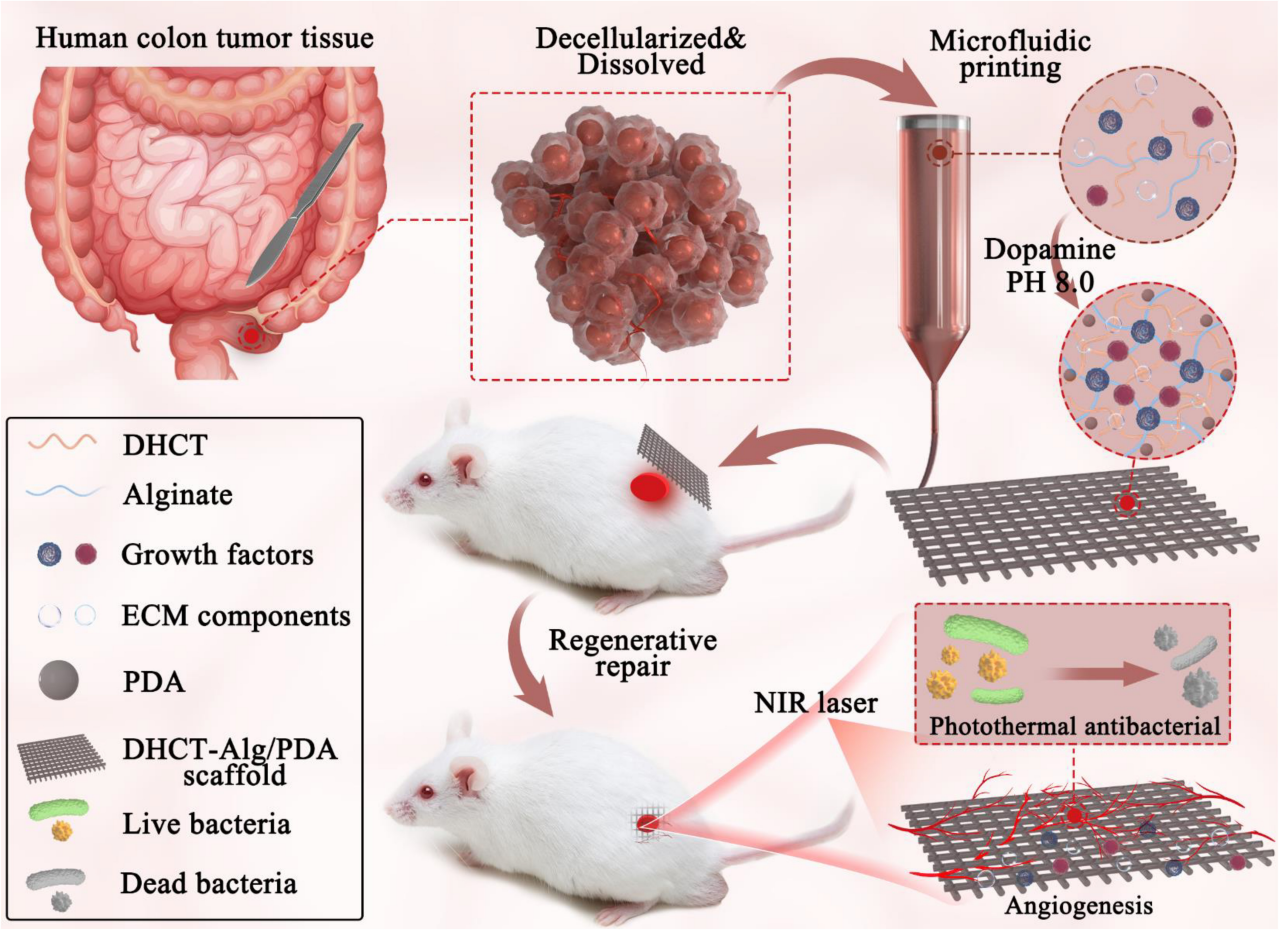
Schematic illustrating microfluidic printing process of DHCT-sodium alginate (Alg)/PDA scaffolds and the application in wound healing.

Here, we propose a decellularized human colon tumor extracellular matrix (DHCT ECM) scaffold with natural polydopamine (PDA) coating for the wound healing application via a microfluidic printing technique (Fig. 1). As a natural polymeric substance inspired by mussels with universal adhesive capabilities, PDA has been widely used for functionalizing biological surfaces [36–38]. The photothermal conversion capability of PDA can impart the modified surfaces with antibacterial abilities because the temperature rise caused by irradiation would destroy the protein structures of microbial and thus kill bacteria [39,40]. Additionally, human colon tumor (HCT) tissue is one of the common biological samples in clinical practice and, interestingly, contain active components that favor angiogenesis and tissue regeneration [41–45]. Thus, they can be utilized as promising tissue engineering matrix after effective decellularization processes owing to their favorable histocompatibility, low immunogenicity, as well as tissue cell induction functions [46,47]. It was thus conceived that the integration of DHCT ECM and PDA can impart the hybrid scaffold with structural support capacity, biocompatibility, angiogenesis, tissue adhesion, as well as antibacterial capabilities that will facilitate wound healing.

For this purpose, we engineered a hydrogel scaffold based on decellularized colon tumor with PDA coating from a microfluidic printing method for bacterial elimination and regenerative repair of acute extensive wounds (Fig. 1). The DHCT ECM was obtained by using a nonionic detergent to remove cells within the colon tumor tissue. Notably, key active components of the ECM, including collagen, fibrin, growth factors, and cytokines, were effectively preserved. By using a microfluidic-integrated 3-dimensional (3D) bioprinting method, we fabricated a tissue-derived hydrogel scaffold based on such a bioactive reagent and then coated it with PDA to achieve desired features. It was worth noting that due to the precise fluid control ability of microfluidics, the porous bioprinting structure could be finely regulated at the micron level. The retained nutrients in DHCT ECM would bring the scaffold about an appealing biocompatibility, cell migration, as well as angiogenesis induction capabilities, while the outstanding photothermal conversion property of the PDA coating imparted the scaffold with a rapid photothermal response and, as a result, an immediate and efficient antibacterial behavior. Specifically, with only 10 min of near-infrared (NIR) irradiation, the scaffold achieved over 90% antibacterial rate. Furthermore, in vivo healing outcomes confirmed excellent wound repair capabilities, especially the promotion of collagen deposition and angiogenesis of the hybrid scaffold. These findings highlighted the values of such tumor tissue-derived materials in wound healing process, which would shed light for the applications of biological tissue-derived materials in regenerative medicine and clinical practice.

## Results and Discussion

**Fig. 2.**
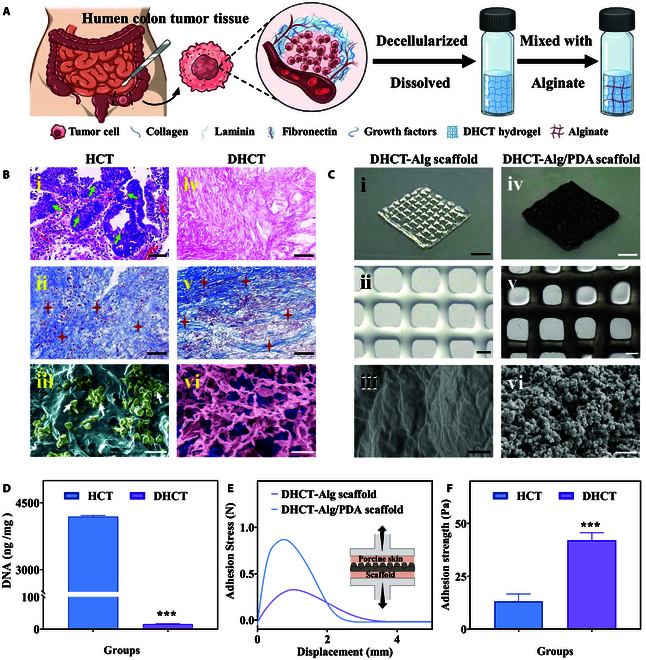
(A) Flow chart of HCT decellularization process. (B) Representative images of HCT and DHCT, including (i and iv) H&E staining, where green arrows indicate HCT cell nuclei, (ii and v) Masson staining, where red stars indicate collagen, and (iii and vi) SEM pseudo-color images, where white arrows point to HCT cells. Scale bars: 100 μm in (i), (ii), (iv), and (v) and 10 μm in (iii) and (vi), respectively. (C) (i) Digital photograph and (ii) optical photograph of DHCT-Alg scaffold; (iii) SEM image of the DHCT-Alg scaffold surface; (iv) digital photograph and (v) optical photograph of DHCT-Alg/PDA scaffold; and (vi) SEM image of PDA coating. Scale bars: 0.5 cm in (i) and (iv), 1,000 μm in (ii) and (v), and 1 μm in (iii) and (vi), respectively. (D) DNA quantification of HCT and DHCT. (E) Stress-displacement curve of the scaffolds. (F) Adhesive strength of the scaffolds. ***P < 0.001.

In a typical experiment, HCT tissue from patients who had not undergone chemotherapy prior to surgery was taken as the source of decellularized scaffolds. After chemical decellularization treatment, the DHCT was dissolved and mixed with sodium alginate (Alg) to form the bioink (Fig. 2A). Notably, the nonionic detergent Triton X-100 was used to effectively remove cytoplasmic and nuclear elements from tumor with little damaging to the bioactive components, and the specimens were then lyophilized for subsequent use (Fig. S1A). It could be seen that without the hemoglobin, the DHCT tissue appeared translucent compared to the original tissue (Fig. S1B). No obvious residual nuclei were found in DHCT samples, indicating that tumor cells had been effectively removed (Fig. 2B, i and iv),whereas key ECM components essential for tissue regeneration, such as fibronectin, type IV collagen, and laminin, were preserved postdecellularization, facilitating tissue repair processes (Fig. 2B, ii and v, and Fig. S2). Additionally, critical growth factors like platelet-derived growth factor, insulin-like growth factor 1, fibroblast growth factors, and transforming growth factor-β were also retained in the DHCT, which might potentially play pivotal roles in wound healing (Fig. S3). Compared to the original tumor tissue, distinctive fibrous structures that support cell adhesion and migration and thus facilitate tissue regeneration could be observed by scanning electron microscopy (SEM) imaging (Fig. 2B, iii and vi). To further quantify the extent of decellularization, we conducted DNA quantification assays and demonstrated that each milligram of tissue contained less than 50 ng of double-stranded DNA (Fig. 2D and Fig. S1C), confirming the thoroughness of the decellularization process. Building upon the results of DNA quantification assays, hematoxylin and eosin (H&E) image comparison, and subsequent in vitro and in vivo experiments, the possibility of immune reactions due to the presence of decellularized residues is minimized, thereby avoiding concerns about the safety of DHCT.

In an effort to enhance the printability of DHCT for scaffold fabrication, we introduced pepsin and acetic acid for further digestion. However, the resulting DHCT solution exhibited low viscosity, which proved to be insufficient for direct printing (Fig. S4A). To address this, Alg was considered to be added because it can offer appropriate yet tunable viscosity favoring the printing process (Fig. S4B). It could be seen that the rheological properties of the DHCT solution was greatly improved with the introduction of Alg, thus enabling the following bioprinting (Fig. S4C). To obtain the designed scaffold based on this bioink, a microfluidic-device-incorporated printing method was utilized (Fig. S5). In the printing process, the bioink was extruded from the microfluidic chip outlet to construct the 3D scaffold. In comparison, it could be found that high concentrations of Alg increased the viscosity of the bioink, which resulted in fibers with smoother surfaces and thus enhanced the printing precision (Fig. S6). With the purpose of achieving scaffolds with satisfactory formability, the final concentrations of DHCT and Alg were optimized and determined to be 2% and 5%, respectively.

Benefitting from the adjustable parameters, the designed 3D architecture of the scaffold can be realized (Fig. 2C, i and ii). These microstructures can also be tuned by changing the orifice of the microfluidic device, the flow rates of the bioink, and the moving speed of the printing platform. Specifically, the large orifice, slow flow rates, and platform moving speed resulted in a large diameter of the fiber and small pore size of the scaffold (Fig. S7). The accompanying swelling of the bioink during printing would further enlarge the fiber diameters. Additionally, based on this flexible printing method, various shapes of scaffolds can be achieved to precisely meet the tissue regeneration demands (Fig. S8). The coating of PDA was carried out in an alkaline environment, where dopamine self-polymerized to form a thin layer and covered the fibers, resulting in the DHCT-Alg/PDA scaffold (Fig. 2C, iv and v, and Fig. S9). Compared to the SEM image of the DHCT-Alg scaffold, which showed relatively smooth surface of the microfiber (Fig. 2Ciii), that of the DHCT-Alg/PDA scaffold demonstrated PDA nanoparticles covering the microfiber (Fig. 2Cvi). The PDA layer then imparted the DHCT-Alg/PDA scaffold with adhesive properties to stick to various material surfaces including plastic, glass, polystyrene, and even biological interface, for example, porcine skins (Fig. S10).

A quantitative study on tissue adhesion ability of the DHCT-Alg/PDA scaffold has also been conducted (Fig. 2E). In the mechanical tests, the scaffolds were adhered to the porcine skin and then stretched vertically until separation. According to the stress-displacement curve, both DHCT-Alg and DHCT-Alg/PDA scaffolds showed typical separation behaviors, despite the relatively large stress the later experienced. The adhesion strengths were analyzed during the stretching test (Fig. 2F). The improved adhesion force demonstrated the benefits of PDA in tissue adhesion, and the maximum adhesion strength was about 3 times that of without PDA. Furthermore, the DHCT-Alg/PDA scaffold adhered to the back skin of a living rat and did not fall off during normal activities (Fig. S11). These findings indicated that the DHCT-Alg/PDA scaffold possessed strong tissue adhesive properties, conferring a significant advantage in wound healing applications.

**Fig. 3.**
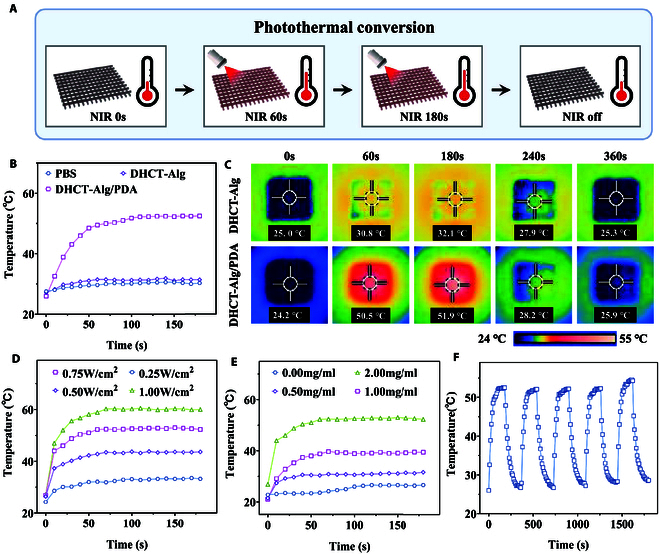
Photothermal performance of the DHCT-Alg/PDA scaffold under NIR irradiation. (A) Schematic diagram of the photothermal conversion study of the scaffold. (B) Recorded temperatures of PBS, DHCT-Alg, and DHCT-Alg/PDA scaffolds under NIR irradiation. (C) Thermal images of DHCT-Alg scaffold and DHCT-Alg/PDA scaffold changing over time under NIR radiation. (D) Temperature changes of 2 mg ml^−1^ DHCT-Alg/PDA scaffold under different laser power densities. (E) Temperature changes of scaffolds with different concentrations of PDA coating, and the NIR irradiation was kept at 0.75 W cm^−2^. (F) Temperature change curve of the DHCT-Alg/PDA scaffold during 5 on/off cycles of NIR irradiation.

The photothermal conversion capability of PDA imparted the DHCT-Alg/PDA scaffold with photothermal responsiveness. To confirm this, the DHCT-Alg/PDA scaffold was irradiated by a NIR beam (808 nm) for 3 min, maintaining a distance of 10 cm between the scaffold and the laser tip, and the temperature was monitored (Fig. 3A). As comparisons, the temperature changes of phosphate buffer saline (PBS), DHCT-Alg scaffold, and DHCT-Alg/PDA scaffold and thermal images of the scaffolds with or without PDA coating were recorded under the conditions that the PDA solution concentration was 2.0 mg ml^−1^, and the laser power density was 0.75 W cm^−2^ (Fig. 3B and C). The temperature of the DHCT-Alg/PDA scaffold exhibited a rapid increase and then stabilized, in contrast to the PBS and DHCT-Alg scaffold groups, where no significant temperature change was evident. Specifically, the effect of PDA concentrations and irradiation intensities on photothermal responsiveness was investigated. It demonstrated that either the strengthened irradiation intensity or the increased PDA concentration would result in the increment in final temperature change (Fig. 3D and E). In detail, under power densities of 0.25, 0.50, 0.75, and 1.00 W cm^−2^ NIR irradiation, the scaffold coated with 2 mg ml^−1^ PDA solution exhibited a rapid temperature rise from 26.9 to 33.2, 43.6, 52.3, and 60.0°C, respectively. Under the same irradiation intensity (0.75 W cm^−2^), the temperature of the scaffold coated with 0, 0.5, 1.0 and 2.0 mg ml^−1^ PDA increased from 21.1 to 26.6, 31.6, 39.4, and 52.3°C, respectively. Moreover, we adjusted the distance between the scaffold and the laser tip and found the temperature of the scaffold increased higher when the distance became smaller (Fig. S12). To avoid damage to the skin caused by excessive temperature while achieving a photothermal responsive antibacterial reaction, the chosen concentration of PDA coating solution was 2.0 mg ml^−1^, and the optimized irradiation intensity was 0.75 W cm^−2^. Furthermore, 5 laser on/off cycles were used to verify the photothermal stability of the DHCT-Alg/PDA scaffold (Fig. 3F). Overall, these results illustrated that the DHCT-Alg/PDA scaffold had efficient and stable photothermal conversion capabilities, laying the foundation for subsequent research.

**Fig. 4.**
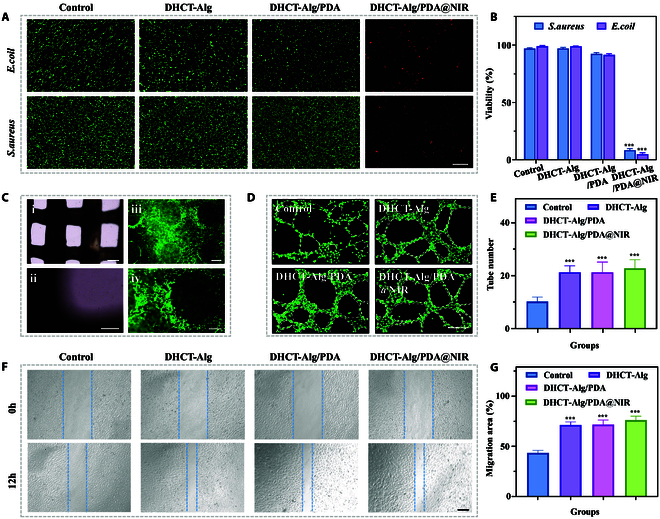
Antibacterial analysis and effects on cell behavior of DHCT-Alg/PDA scaffolds. (A) Fluorescent images depicting *S. aureus* and *E. coli* in in various experimental groups. Scale bar: 200 μm. (B) Quantitative bacterial viability in various treatment groups. (C) Representative images of NIH 3T3 after 3 d of adhesion to DHCT-Alg/PDA scaffold surface, including (i and ii) optical image and (iii and iv) live/dead staining. Scale bars: 500 μm in (i) and (iii), 250 μm in (ii) and (iv). (D) Representative images of angiogenesis in various groups. Scale bar: 200 μm. (E) Quantitative analysis of angiogenesis assay. (F) In vitro scratch measurement, where the dotted lines indicate the scratch edge. Scale bar: 300 μm. (G) Quantitative analysis of in vitro scratch results. ***P < 0.001.

The sensitive photothermal reactivity of the scaffold can thus provide sufficient heat to change the protein structures and disrupt bacterial membranes, further contributing to the bacterial eradication. It could be seen from the live/dead staining of bacteria that the 0.75 W cm^−2^ NIR irradiation heated the DHCT-Alg/PDA scaffold up to about 50°C within 10 min, causing a significant antibacterial effect (Fig. 4A). As a comparison, the control (without any scaffold) group, DHCT-Alg group, and DHCT-Alg/PDA were also cocultured with both *Staphylococcus aureus* (*S. aureus*) and *Escherichia coli* (*E. coli*). Since the DHCT-Alg/PDA@NIR group exhibits excellent antibacterial effect under NIR irradiation, the image showed mostly red fluorescence and a small amount of green fluorescence, whereas the remaining groups showed mostly green fluorescence. Quantitatively, the survival rates of *S. aureus* and *E. coli* dropped to approximately 8% and 4%, respectively, in the DHCT-Alg/PDA under the NIR group, demonstrating the NIR-responsive antimicrobial property (Fig. 4B).

Before investigating the tissue regeneration ability of the DHCT-Alg/PDA scaffold, its biocompatibility was studied at the in vitro level. It could be seen that the fibroblast cells (NIH 3T3) seeded on the surface of the scaffold showed good viability after 3 d of cultivation (Fig. 4C). In detail, cells showed similar viabilities compared with other groups, which could be inferred from the live/dead staining and results based on the cell counting kit-8 assay for 1 d (Fig. S13A and B). The DHCT-Alg/PDA scaffold also showed its ability to promote cell migration. Based on the scratch test, all of the DHCT-Alg, DHCT-Alg/PDA, and DHCT-Alg/PDA@NIR (DHCT-Alg/PDA under NIR irradiation) groups showed accelerated wound closure within 12 h compared to the control group, demonstrating the bioink’s promotion on cell migration (Fig. 4F and G). Moreover, due to the ECM preserved in DHCT, the scaffold could also show angiogenic capacity (Fig. 4D). It could be seen that more vascular-like tubes formed in the DHCT-Alg group, DHCT-Alg/PDA group, and DHCT-Alg/PDA@NIR group relative to the control group (Fig. 4E). Altogether, the constructed scaffold exhibited good biocompatibility, angiogenesis ability, and in vitro tissue regeneration potential, all of which are advantageous for promoting wound healing in vivo.

**Fig. 5.**
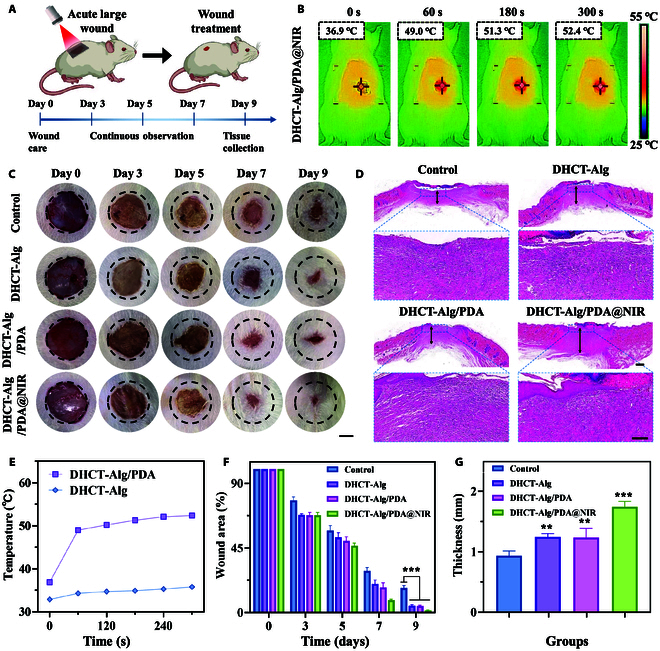
Wound healing in the acute large-area full-thickness skin defect model. (A) Schematic diagram of wound healing procedure. (B and E) Photothermal images and statistical analysis of acute large-area wounds after DHCT-Alg/PDA@NIR treatment. (C and F) Photographs of rat back wounds treated with various strategies and quantitative assessment of wound areas. Scale bar: 0.5 cm. (D and G) H&E evaluation and quantitative assessment of the granulated tissue thickness on wounds on day 9. Scale bars: 500 μm (top) and 200 μm (bottom). ***P < 0.001, **P < 0.01.

To explore the practical wound healing effect of the scaffolds, we constructed a full-thickness skin defect model with an acute large-area circular wound (1.5 cm in diameter) on the dorsal region of rats (Fig. 5A). According to the treated materials, the rats were divided into control (without any scaffold), DHCT-Alg, DHCT-Alg/PDA, and DHCT-Alg/PDA@NIR groups (Fig. S14). Among them, the temperature of the DHCT-Alg/PDA@NIR group was monitored via thermal imaging (Fig. 5B). It was found that the temperature of the wound increased to about 50°C within 1 min to release the antibacterial ability. During the irradiation, the wound temperature remained almost unchanged after 5 min, which guaranteed the sustainable antibacterial reaction (Fig. 5E). The wound closure processed of different groups were observed and recorded on days 0, 3, 5, 7, and 9 after treatment (Fig. 5C). It was found that the DHCT-Alg/PDA@NIR group exhibited the most rapid healing speed compared with others. On day 9, the unclosed wound area of the rats in the DHCT-Alg/PDA@NIR group was only 1.3%, while those of other groups were 16.9%, 4.8%, and 4.4%, respectively (Fig. 5F). This enhancement could likely be ascribed to the NIR-responsive antibacterial efficacy conferred by the PDA coating on the scaffold. H&E staining further examined wound healing status in each group (Fig. 5D). It could be seen that after 9 d of treatment, despite that new granulation tissues could be found in the DHCT-Alg group, DHCT-Alg/PDA group, and DHCT-Alg/PDA@NIR group, the thickness of that in the DHCT-Alg/PDA@NIR group exceeded (Fig. 5G). These results preliminarily demonstrated the improved wound healing property of the resultant scaffold with a NIR-dependent antibacterial property.

**Fig. 6.**
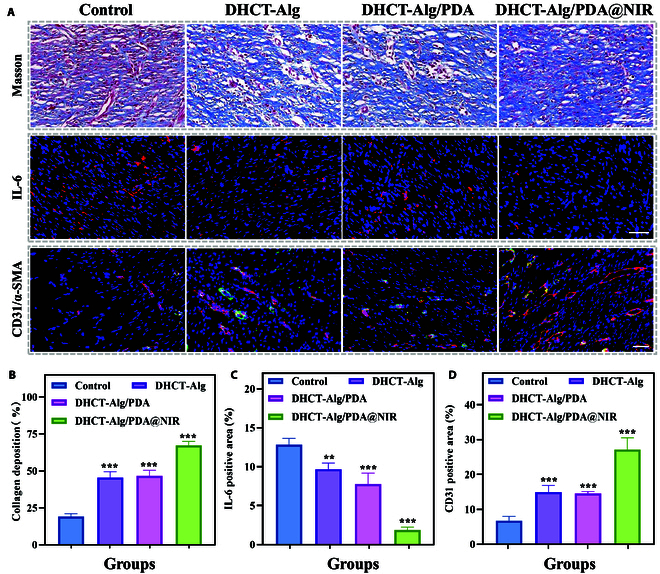
Collagen deposition, inflammation, and angiogenesis analyses in acute large-area wounds. (A) Masson staining and immunofluorescence of IL-6 (red) and CD31 (red) and a-SMA (green) in wound samples. Scale bars: 100 μm (top) and 50 μm (middle and bottom). (B to D) Quantification assessment of (B) collagen deposition, (C) IL-6 expression, and (D) CD31 expression. **P < 0.01, ***P < 0.001.

The wound regeneration process was also deeply analyzed. The collagen deposition at the wound site was characterized by Masson staining. It could be seen that the largest amount of collagen deposition was found in the DHCT-Alg/PDA@NIR group, suggesting facilitated wound healing (Fig. 6A and B). In addition, immunohistochemistry on day 9 evaluated interleukin-6 (IL-6) expression. The down-regulated expression of DHCT-Alg/PDA@NIR group indicated the antibacterial and anti-inflammatory effects of DHCT-Alg/PDA compared to the control group, where severe inflammation took place (Fig. 6A and C). Moreover, in vivo angiogenesis was evaluated through dual-immunofluorescence staining targeting vascular endothelial cell markers CD31 and α-smooth muscle actin (α-SMA). Their lower expression in the control group suggested a lower level of vascularization (Fig. 6A and D). Compared with the DHCT-Alg/PDA group, the expression level of CD31 and α-SMA were elevated in the DHCT-Alg/PDA@NIR group due to the reduced inflammatory response resulting from bacterial infection. Taken together, these findings suggest that the DHCT-Alg/PDA scaffold effectively facilitates tissue remodeling, reduces inflammation, and promotes angiogenesis, with no obvious immune damage, thereby promoting acute large-area wound healing.

## Conclusion

In summary, we proposed a decellularized colon tumor-derived ECM hydrogel scaffold by microfluidic printing for wound healing. These innovative scaffolds were designed to preserve essential components of tissue ECM like growth factors, collagen, and fibrin, all of which played pivotal roles in tissue regeneration. The matrix, in turn, caused a substantial promotion in the healing process of acute large-area wounds. Benefiting from the precise and flexible control of microfluidic printing process, the designed morphology could be realized to fit different wounds. Furthermore, the scaffolds were coated by dopamine to acquire photothermal-induced antibacterial properties when subjected to NIR irradiation. This unique feature contributed to a significant reduction in tissue inflammatory reactions, ultimately expediting the repair and regeneration of damaged tissues. This research not only verifies the effectiveness of DHCT-ECM-derived hydrogel scaffolds in acute large-wound healing but also opens an avenue for tissue-derived materials in tissue regeneration field. However, the application of tumor tissue-derived hydrogels to human wounds still raises concerns, including possible safety and ethical issues with the source of the samples and the presence of unknown carcinogens. In addition, hydrogel material preparation processes are difficult to standardize and may degrade or deteriorate during storage. Even so, we believe in the potential of natural tumor extracts as promising candidates in tissue repair.

## Materials and Methods

### Materials

Sodium alginate and calcium chloride were procured from Sigma-Aldrich. Dopamine hydrochloride was obtained from Macklin Shanghai. Propidium iodide (PI) and SYTO were obtained from KeyGEN BioTECH (Jiangsu, China). Cell lines were supplied by Wenzhou Institute, University of Chinese Academy of Sciences (WIUCAS). The IL-6 and α-SMA antibodies were provided by Boster Biological Technology (Wuhan, China). CD31 antibody was purchased from Abcam Plc. The experimental rats were sourced from Zhejiang Vital River Laboratory Animal Technology Co., Ltd. Animal experimental ethics was approved by the Animal Ethics Committee of WIUCAS (WIUCAS23071303).

### Characterization

Bright-field microscopic images were acquired through an Olympus microscope. Images of bacterial and cells staining were acquired using an Axio Vert A1 fluorescence microscope. SEM imaging was conducted using SU8010, Hitachi.

### Decellularization of HCT tissue

All studies were performed according to recognized ethical guidelines (2023-K-247-02) approved by the Ethics Committee of the Second Affiliated Hospital and Yuying Children’s Hospital, Wenzhou Medical University. Fresh HCT samples with a radius and thickness of approximately 0.5 cm were obtained from the operating room. The specimens were rinsed with PBS containing penicillin and streptomycin and then underwent a series of treatments, including 1% (v/v) Triton X-100 solution and 0.1% (v/v) ammonium hydroxide. The prepared DHCT was dissolved using a solution of pepsin (1%) and acetic acid (100 mM).

### DNA content

Nuclear 4’,6-diamidino-2-phenylindoleI staining was conducted on DHCT to assess the presence of residual nuclei. Additionally, DNA content analysis was carried out by extracting DNA from both HCT and DHCT by a commercial DNA kit (KeyGEN BioTECH). Quantitative measurements of extracted DNA were performed using a spectrophotometer following the provided manual, and DNA concentration was expressed as nanogram per milligram of dry tissue weight.

### Fabrication of DHCT-Alg/PDA scaffold

Firstly, disinfected the printing device with UV light for 4 h. 3ds Max software was used to design different shapes and structures of the scaffold models. The prepared DHCT (2% and 4%) was thoroughly mixed with Alg (1%, 2%, 3%, 4%, and 5%), fully dissolved to form the bioink, and stored overnight before printing at room temperature. Dopamine could self-polymerize in alkaline solutions to form PDA. Therefore, after printing, the DHCT-Alg scaffold was soaked in the PDA solution for 24 h to be coated with PDA layers on the surface.

### Adhesion experiments

The 1.5 cm × 1.5 cm scaffold was attached to the horizontally fixed pig skin with a preapplied pressure of 5 N for 2 min. Detachment was manually stopped upon separation of the scaffold from the skin tissues. Maximum adhesion was determined from stress-displacement curves to assess scaffold adhesion ability.

### Photothermal properties of DHCT-Alg/PDA scaffold

DHCT-Alg scaffold and DHCT-Alg/PDA scaffold were exposed to NIR irradiation (808 nm, 3 min). We kept the distance between the scaffold and the laser tip at 10 cm and used an infrared thermal imaging camera to record the temperature changes of the scaffold (FLIR E5-XT, Germany).

### Antibacterial efficacy assessment

The 2 types of bacterial suspensions were diluted to 2 × 10^6^ CFU/ml. About 1 ml of each bacterial group was dropped into the wells of a 24-well plate and incubated with the scaffolds. For the DHCT-Alg/PDA@NIR group, the scaffold was exposed with a 0.75 W cm^−2^ NIR irradiation for 10 min, when the laser tip was 10 cm away from it. The temperature of the DHCT-Alg/PDA scaffold rapidly rose so that the scaffold could exhibit photothermal responsive antibacterial properties. The bacteria after different treatments were stained with SYTO and PI for 20 min, and then 50 μl of the mixture was extracted and subsequently examined under a fluorescence microscope.

### Biocompatibility test

First, we inoculated NIH 3T3 cells (2 × 10^3^ cells per well) and waited for cells to adhere to the plates. Following that, the culture medium was substituted with 200 μl of filtrate from DHCT-Alg scaffold and DHCT-Alg/PDA scaffold, respectively. In the control group, the original culture medium was used. The DHCT-Alg/PDA@NIR group was additionally treated with NIR (808 nm, 3 min) treatment. After incubating for 1 d, cell counting kit-8 solution was added, and the absorbance was measured after 4 h. Meanwhile, 1.0 μl of calcein-AM (Molecular Detection Company) and PI was added to the culture media to stain viable cells, and the live/dead staining fluorescence images were obtained from an inverted fluorescence microscope.

### Cell adhesion assay

A total of 2 × 10^5^ NIH 3T3 cells were seeded on DHCT-Alg/PDA scaffolds and cultured at 37°C, 5% CO_2_. Cells adhering to the scaffold surface were observed by live/dead staining fluorescence images after 72 h of incubation.

### Scratch wound healing assay

One milliliter of HUVEC (2 × 10^5^ cells) suspension was placed in a 24-well plate. Subsequently, we gently scraped the cell monolayer using a p200 pipette and removed unattached cells with PBS. The filtrate from the DHCT-Alg/PDA scaffold was subsequently added to the well for cell culture, and observations were made at appropriate time points.

### Matrigel tube formation assay

Before seeding HUVECs (5 × 10^4^ cells per well), Matrigel matrix was applied to coat the 24-well plates (Matrigel: ECM = 1:1, 250 μl per well). After the cells adhered to the wall, the cell culture media were replaced by leachates of DHCT-Alg/PDA scaffold. Human umbilical vein endothelial cells were stained with calcein-AM, and tube formation was observed under a fluorescence microscope after 6 h of coculture.

### Animal experiments

In order to evaluate the effect of DHCT-Alg/PDA scaffold on wound repair, an acute large-area full-thickness wound model was established in 24 healthy Sprague-Dawley (SD) rats, randomly divided into 4 groups: PBS, DHCT-Alg scaffold, and DHCT-Alg/PDA scaffold. Following anesthesia, the dorsal surfaces of the rats were shaved, and large circular full-thickness skin wounds (1.5 cm in diameter) were made on their backs. Afterward, the scaffolds prepared via the aforementioned method was gently placed on the wounds. Additionally, the DHCT-Alg/PDA@NIR group received NIR irradiation (808 nm, 0.75 W cm^−2^, 10 cm high) for approximately 10 min. Wound photographs were captured on days 0, 3, 5, 7, and 9. Granulation tissue, along with surrounding tissue, was harvested and preserved in formaldehyde. Subsequently, tissue samples were stained by H&E, Masson’s trichrome, and immunofluorescence.

### Statistical analysis

In the analysis process, in order to facilitate statistics, GraphPad Prism 9 was used to analyze the significant differences, the mean ± standard deviation. Student *t* test was used for the analysis, and the significance was expressed as *P < 0.05, **P < 0.01, ***P < 0.001.

## Data Availability

All data needed to evaluate the conclusions in the paper are present in the paper or the Supplementary Materials. Additional data related to this paper may be requested from the authors.
