# Supplementary material for: Decellularized Tumor Tissues Integrated with Polydopamine for Wound Healing
Source: Research (Wash D C). 2024 Aug 15;7:0445. doi: 10.34133/research.0445 (PMC11301524; doi:10.34133/research.0445)
Supplement: Supplementary 1 — Figs. S1 to S14 [file research.0445.f1.docx]

**Supplementary**

**Decellularized tumor tissues integrated with polydopamine for wound healing**

Hongzheng Li ^a^, Xiang Lin ^b,c^, Shangrui Rao ^a^, Gongting Zhou ^a^, Letian Meng ^a^, Yunru Yu ^c,^*, Jinglin Wang ^b,^*, Xiaolei Chen ^d,^*, Weijian Sun ^a,^*

^a^ Department of Gastrointestinal Surgery, The Second Affiliated Hospital of Wenzhou Medical University, Wenzhou, 325027, China

^b^ Division of Hepatobiliary and Transplantation Surgery, Department of General Surgery, Nanjing Drum Tower Hospital, the Affiliated Hospital of Medical School, Nanjing University, Nanjing, 210008, China

^c^ Pharmaceutical Sciences Laboratory, Åbo Akademi University, Turku, 20520, Finland

^d^ Department of Gastrointestinal Surgery, The First Affiliated Hospital of Wenzhou Medical University, Wenzhou, 325027, China

Email: [yunru.yu@abo.fi](mailto:yunru.yu@abo.fi) (Y. Y); [cw20120817@163.com](mailto:cw20120817@163.com) (J. W); chenxiaolei0577@126.com (X. C); [fame198288@126.com](mailto:fame198288@126.com) (W. S)


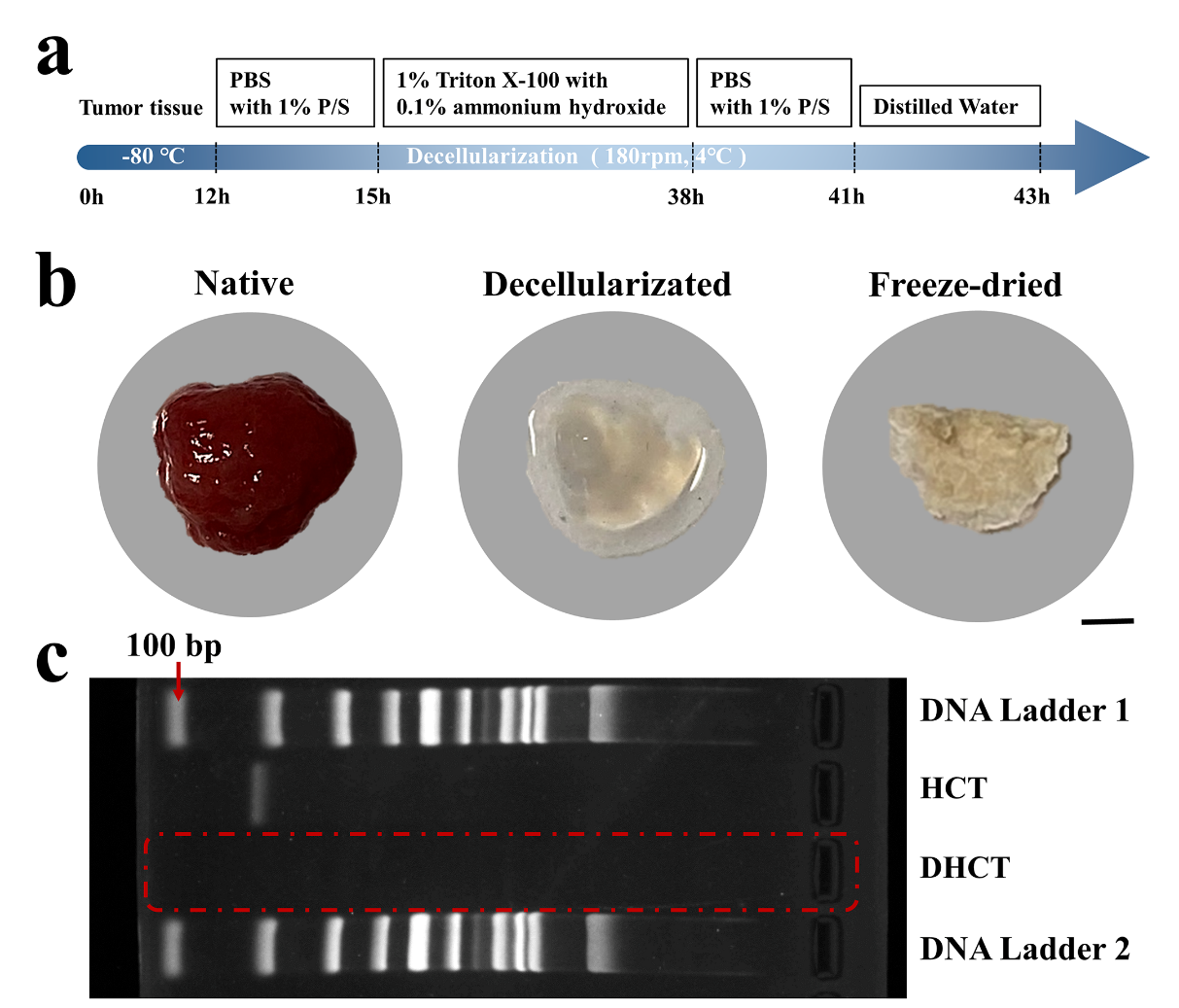


**Figure S1.** (a) Decellularization scheme for HCT; (b) Representative images of native HCT, DHCT and freeze-dried DHCT. Scale bar: 250 mm; (c) dsDNA fragments in HCT and DHCT (detected by 1.0% agarose gel).


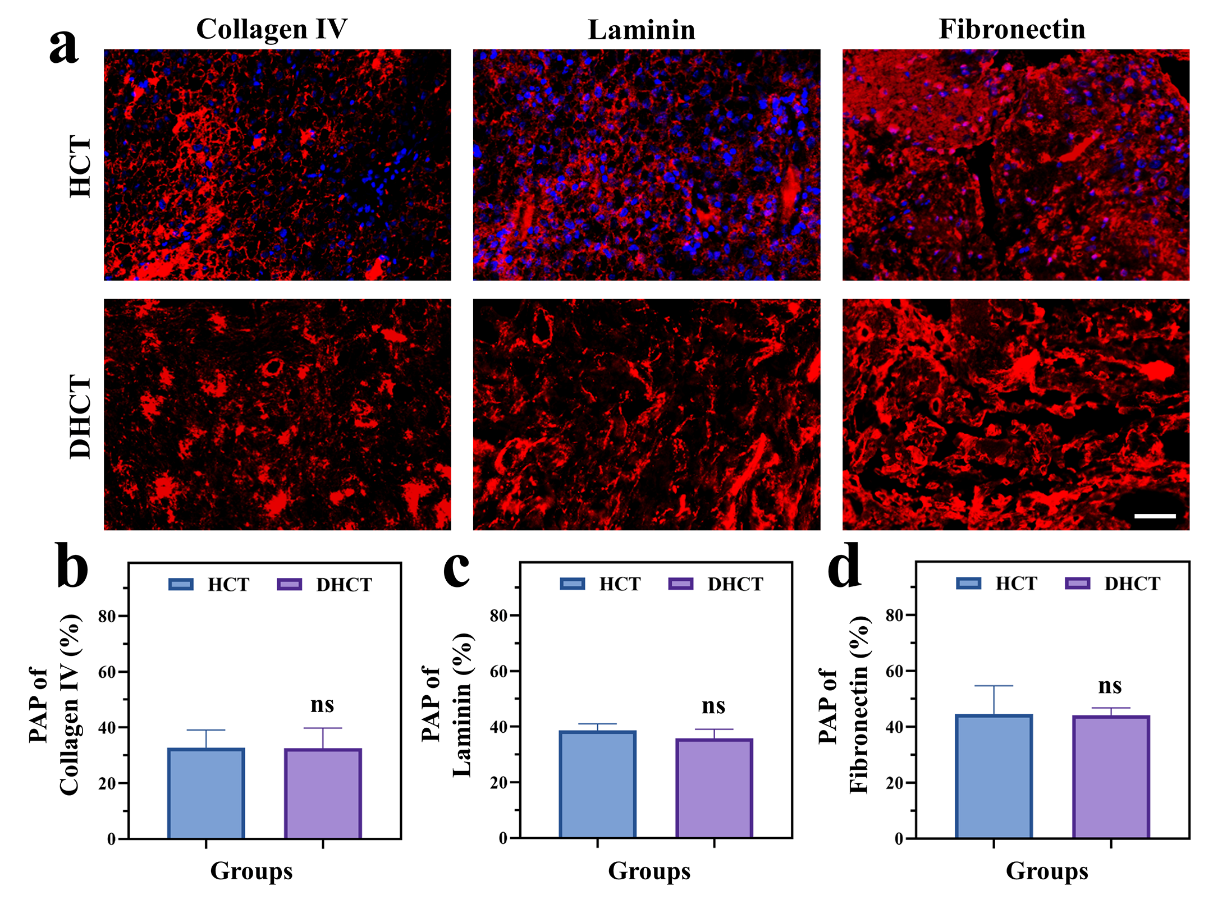


**Figure S2.** (a) Representative immunofluorescence images of HCT and DHCT samples, showing type IV collagen, laminin and fibronectin. Scale bar: 100 μm. (b-d) Statistic analysis of positive area percentage (PAP) representing collagen IV, laminin, and fibronectin respectively. Ns. no significant difference.


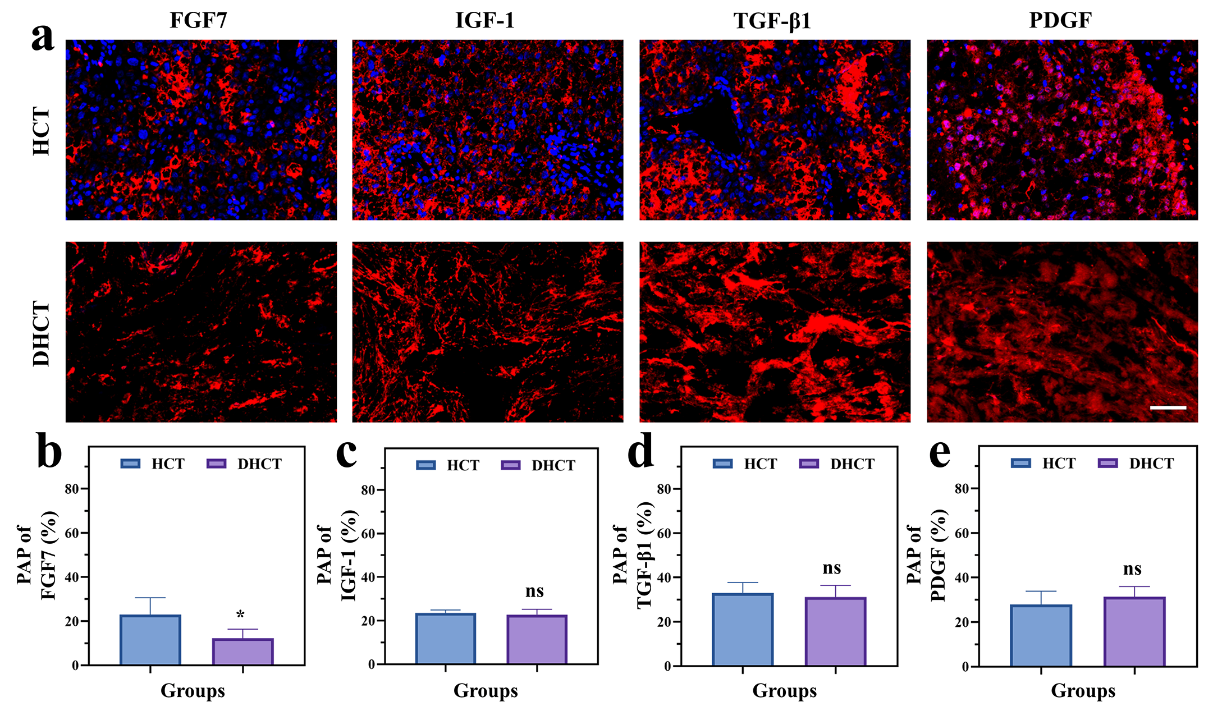


**Figure S3.** Representative fluorescence images of growth factors before and after decellularization, including FGF7, IGF-1, TGF-β1 and PDGF, Scale bar: 100 μm. (b-e) Statistic analysis of PAP of FGF7, IGF-1, TGF-β1, and PDGF respectively. *p< 0.05, ns. no significant difference.


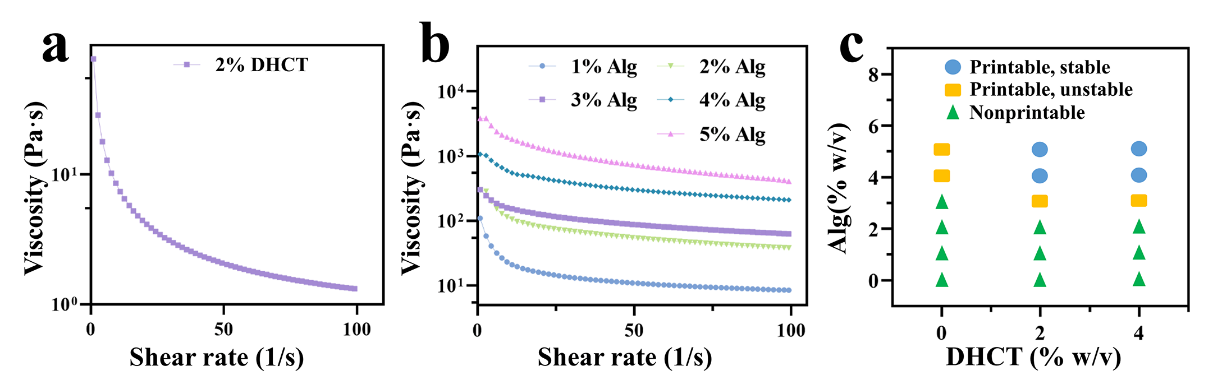


**Figure S4.** (a) Viscosity curves of 2% DHCT solution under various shear rates; (b) Viscosity curves of Alg solution under various shear rates; (c) Printability assessment of diverse bioinks with varied Alg/DHCT compositions.
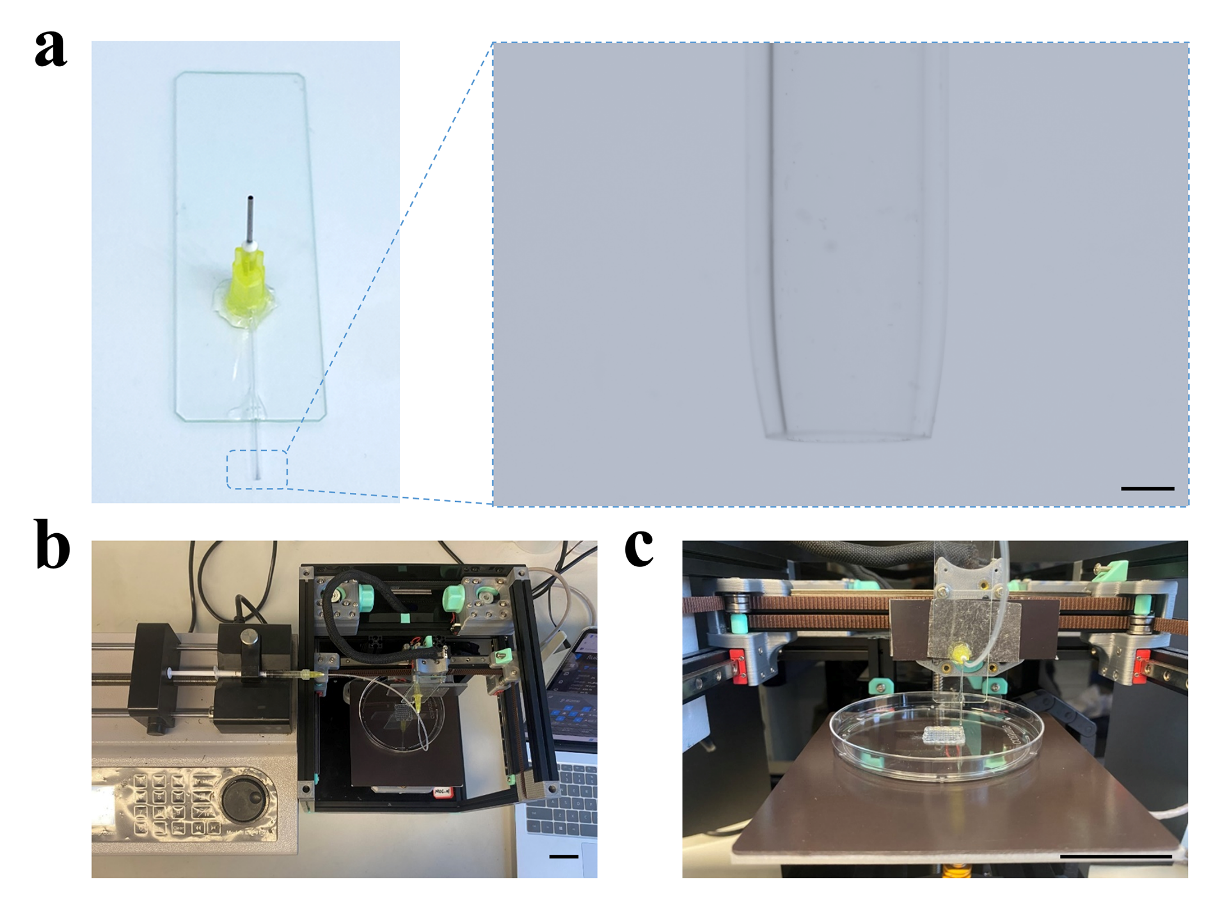


**Figure S5.** (a) Digital images of the microfluidic chip. Scale bar: 100 μm; (b) An overhead view of the microfluidic printing device; (c) Front view of microfluidic printing process. Scale bar: 3cm.


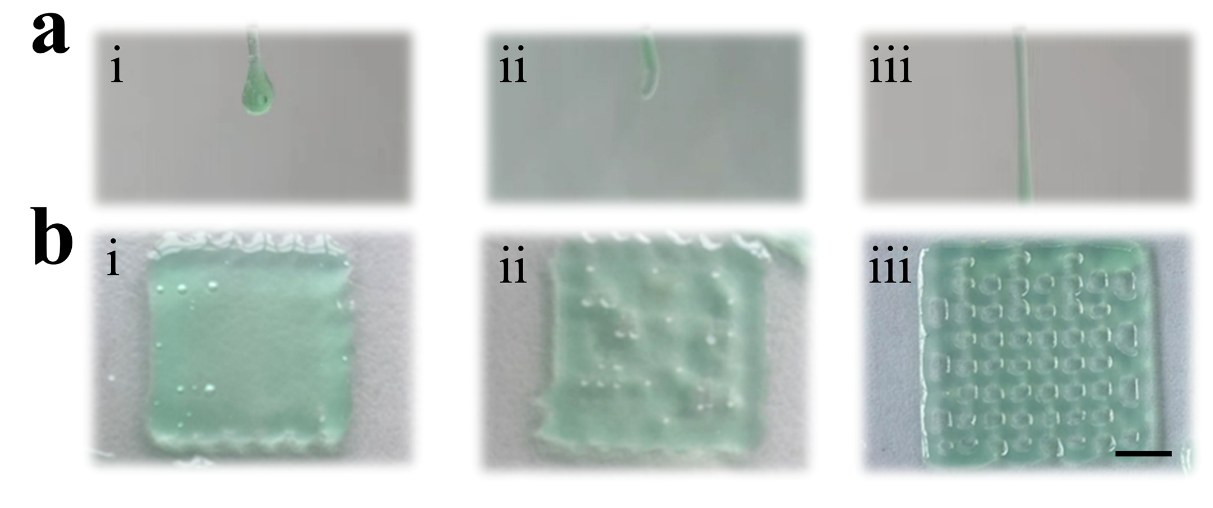


**Figure S6.** (a) Extrusion process of (i) 2%/1% DHCT/Alg, (ii) 2%/3% DHCT/Alg, and (iii) 2%/5% DHCT/Alg; (b) DHCT/Alg scaffolds printed by (i) 2%/1% DHCT/Alg, (ii) 2%/3% DHCT/Alg, and (iii) 2%/5% DHCT/Alg. Scale bar: 0.5cm.


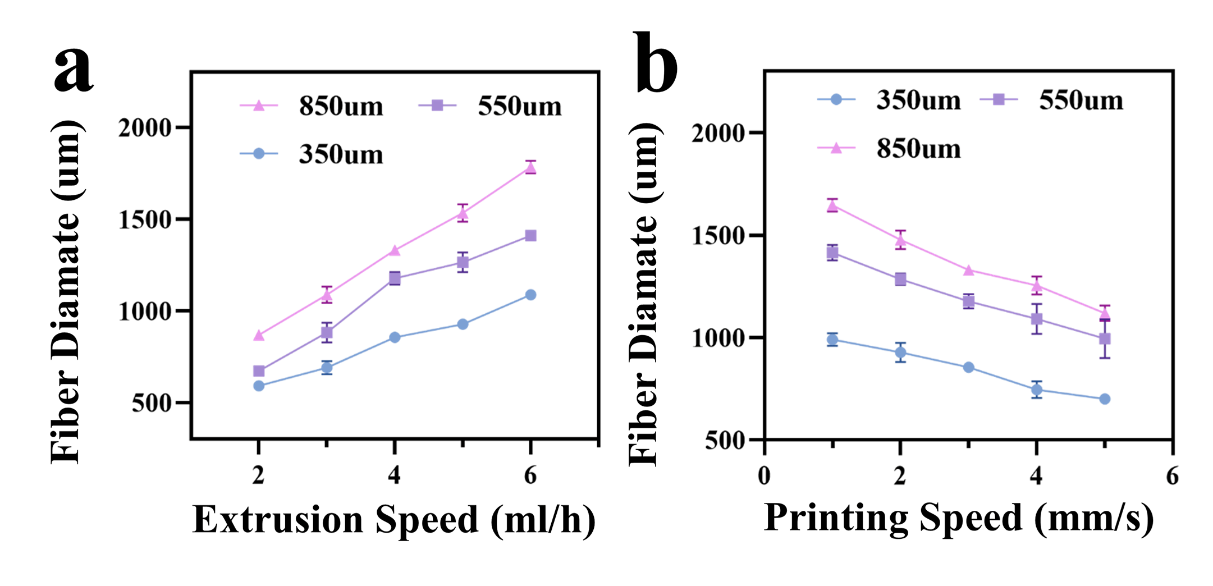


**Figure S7.** (a) The relationship between the fiber diameter and the extrusion speed using different orifices of microfluidic chips. (b) The relationship between the fiber diameter and printing speed.


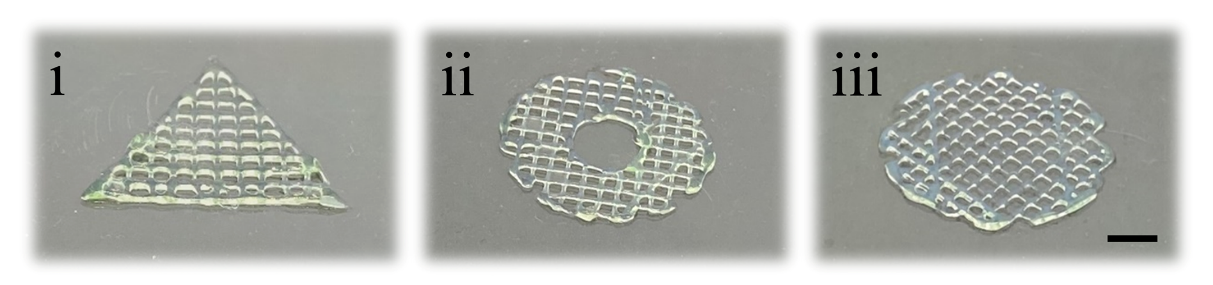


**Figure S8.** Optical images of the printed scaffolds with (a) triangular bracket, (b) hollow circle, and (c) gear. Scale bar: 0.5 cm.


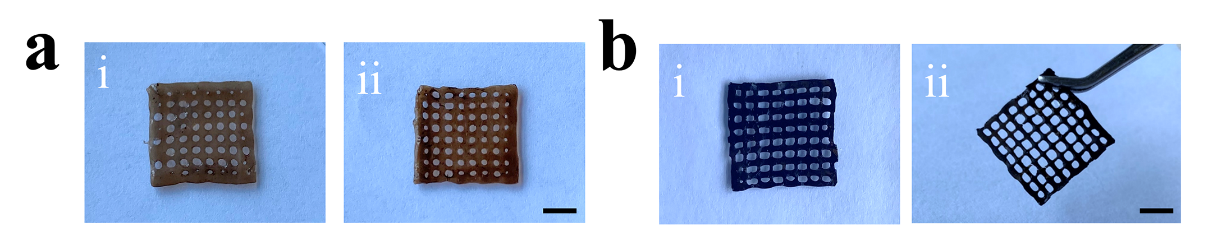


**Figure S9.** (a) Optical images of scaffolds coated with (i) 0.5mg ml^-1^, and (ii) 1mg ml^-1^ PDA solutions; (b) Optical image of 2mg ml^-1^ DHCT-Alg/PDA scaffold (i) placed on the table, and (ii) picked up by a tweezer. Scale bar: 0.5cm.


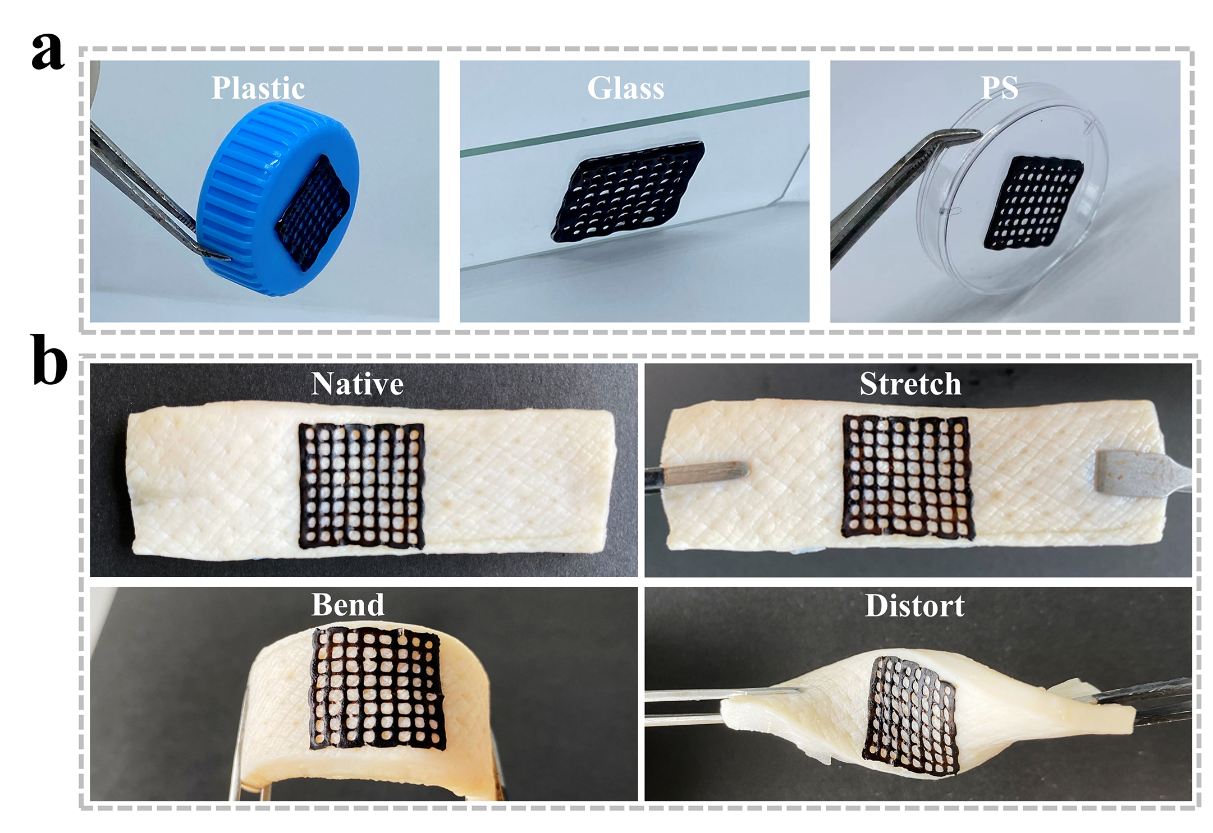


**Figure S10.** Adhesion test of DHCT-Alg/PDA scaffold. (a) Images of scaffold adhered to different materials including plastic, glass, and polystyrene (PS); (b) Images of scaffold adhered to porcine skin before and after bending and deformation.


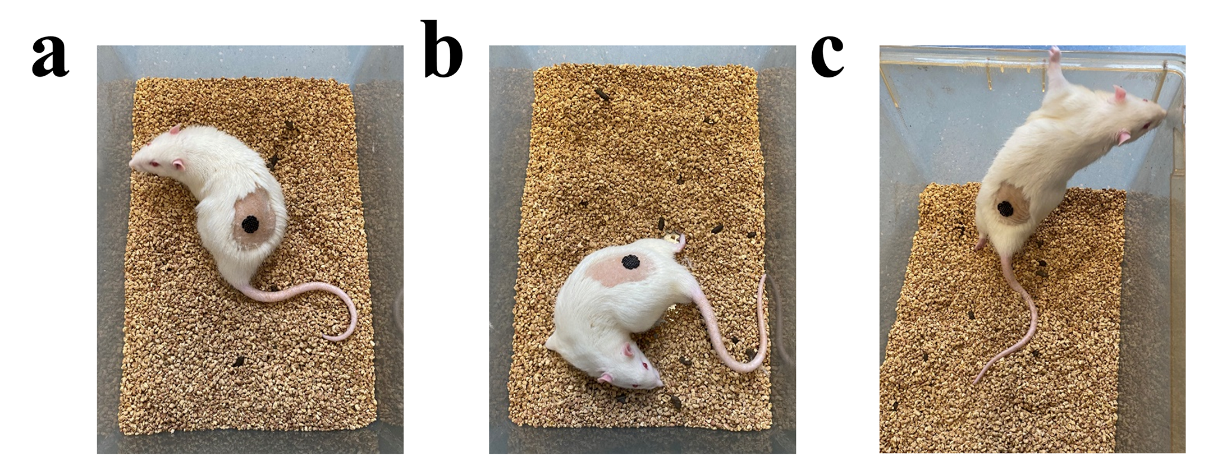


**Figure S11.** Optical images of the DHCT-Alg/PDA scaffold attached to a living rat with different behaviors.


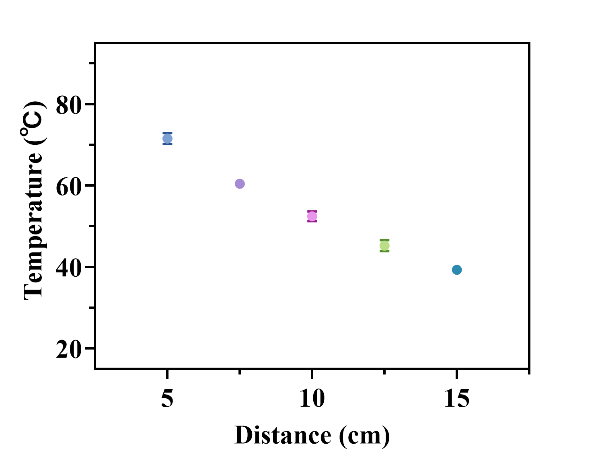


**Figure S12.** Temperature changes of DHCT-Alg/PDA scaffold at different laser tip distances.


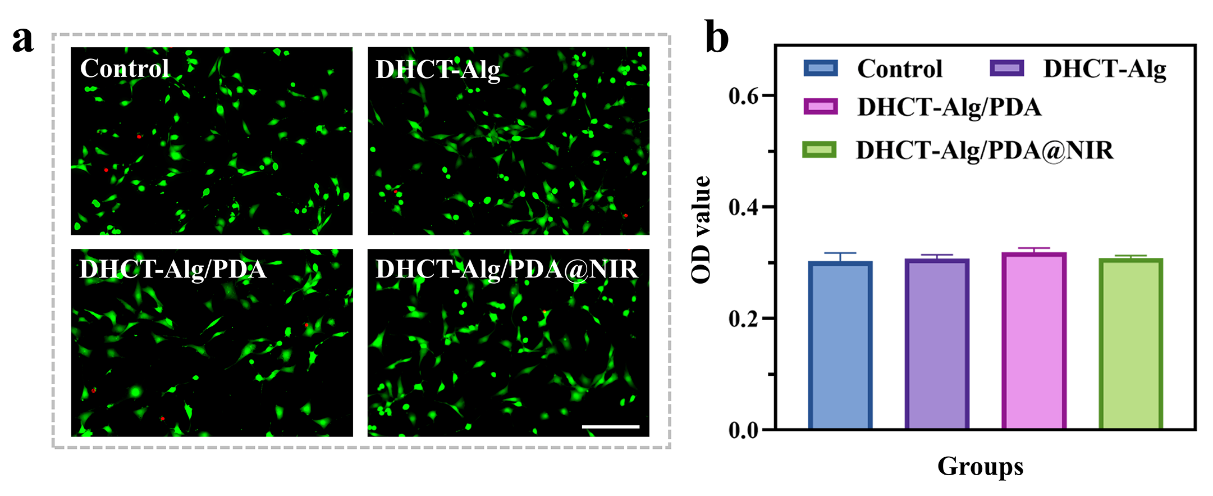


**Figure S13.** (a) Fluorescence images of NIH 3T3 cells 1 day after different treatments. Scale bar: 200 μm; (b) OD value of NIH 3T3 cells.


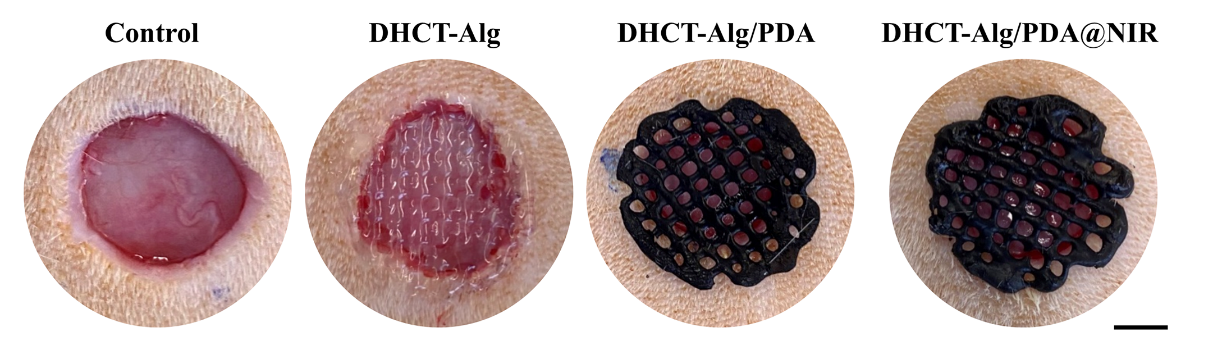


**Figure S14.** Digital images of the large-area wound sites with different treatments. Scale bar: 500 μm.
